# Supplementary material for: SNX19 restricts endolysosome motility through contacts with the endoplasmic reticulum
Source: Nat Commun. 2021 Jul 27;12:4552. doi: 10.1038/s41467-021-24709-1 (PMC8316374; doi:10.1038/s41467-021-24709-1)
Supplement: Supplementary file 1 — Supplementary Information [file 41467_2021_24709_MOESM1_ESM.docx]

**Supplementary Information for:**

**SNX19 restricts endolysosome motility through contacts with the endoplasmic reticulum**

Amra Saric^1^, Spencer A. Freeman^2^, Chad Williamson^1^, Michal Jarnik^1^, Carlos M. Guardia^1^, Michael S. Fernandopulle^3^, David C. Gershlick^1,4^ & Juan S. Bonifacino^1*^

^1^ Neurosciences and Cellular and Structural Biology Division, *Eunice Kennedy Shriver* National Institute of Child Health and Human Development, National Institutes of Health, Bethesda, Maryland, USA.

^2^ Program in Cell Biology, Peter Gilgan Centre for Research and Learning, Hospital for Sick Children, Toronto, ON, Canada.

^3^ Neurogenetics Branch, National Institute of Neurological Disorders and Stroke, National Institutes of Health, Bethesda, Maryland, USA

^4^ Current address: Cambridge Institute for Medical Research, University of Cambridge, Cambridge, UK

*Corresponding author

juan.bonifacino@nih.gov

**
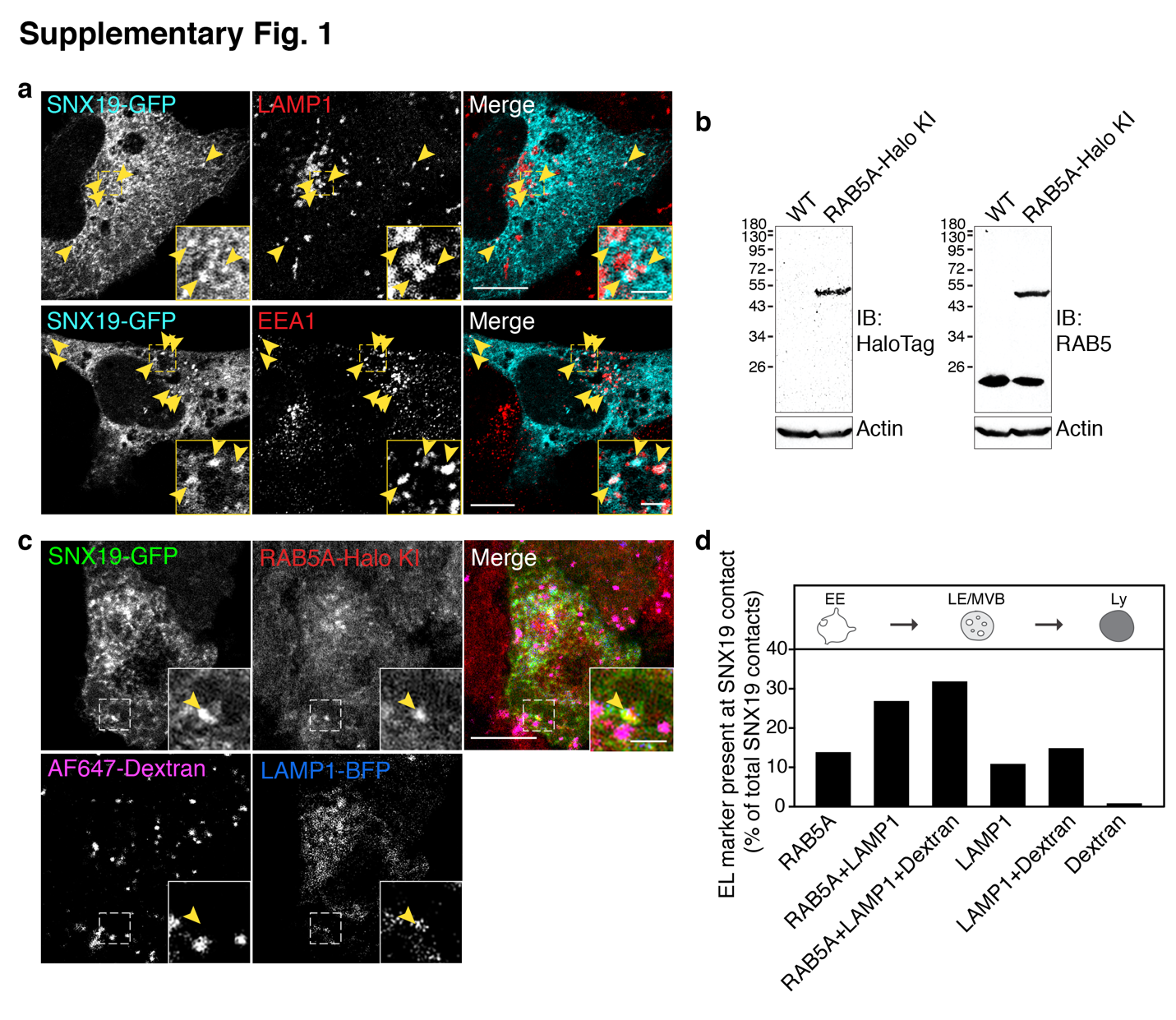
**

**Supplementary Fig. 1: Endolysosomal markers at SNX19-positive contacts. a,** U-2 OS cells were transfected with a plasmid encoding SNX19-GFP, fixed and immunostained with antibodies to the endogenous late-endosomal/lysosomal marker LAMP1 or the early endosomal marker EEA1, and imaged by confocal microscopy. Arrowheads point to SNX19-GFP-EL contacts. **b,** Whole cell lysates of WT and RAB5A-Halo-KI HeLa cells were run on SDS-PAGE and immunoblotted with anti-Halo Tag and anti-RAB5 antibodies. The positions of molecular mass markers (in kDa) are indicated on the left. **c,** Live-cell imaging of RAB5A-Halo-KI HeLa cells co-transfected with plasmids encoding SNX19-GFP and LAMP1-BFP and allowed to endocytose Alexa Fluor 647 (AF647)-Dextran overnight followed by a 2-h chase prior to imaging by confocal microscopy. Endogenous RAB5A was visualized in the red channel by labeling of Halo Tag with Janelia Fluor 549 cell-permeable dye. Insets are magnified views of the boxed areas. As an example, the arrowhead points to a structure having SNX19-GFP, RAB5-Halo and LAMP1-BFP, but not AF647-Dextran (*i.e.*, an endosome). **d,** RAB5A-Halo-KI HeLa cells treated as in **c** were imaged live by confocal microscopy and the percentages of all SNX19-GFP contacts that contained the indicated EL markers (*x*-axis) were plotted. Indicated across the top is a general scheme of the EL compartments containing the marker(s) on the *x*-axis. EE, early endosome; MVB, multivesicular body; LE, late endosome; Ly, lysosome. Data are the same as those plotted in the Venn diagram in Fig. 1h. A total of 135 contacts across 2 experiments, 15-20 cells per experiment, were quantified. Source data are provided as a Source Data file. Scale bars: 10 μm, 2 μm insets.


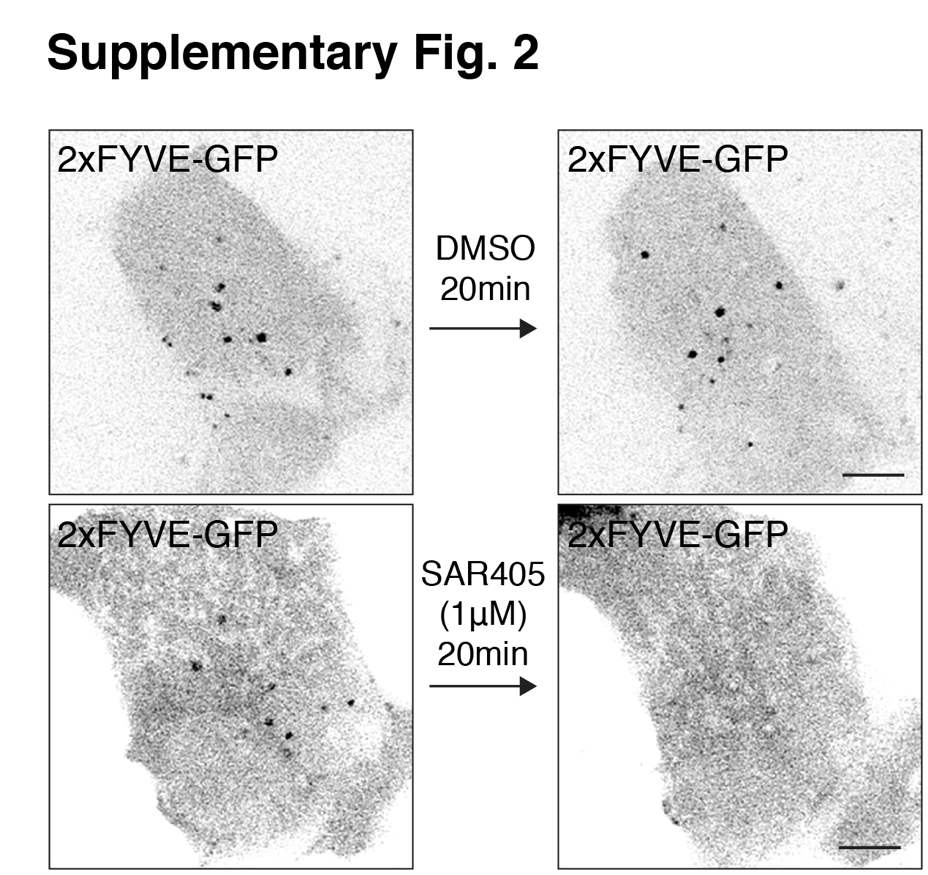


**Supplementary Fig. 2: SAR405 inhibits PI(3)P synthesis.** U-2 OS cells transfected with a plasmid encoding the PI(3)P probe 2xFYVE-GFP were treated with either DMSO (vehicle control) (top panels) or 1 μM SAR405 (bottom panels) for 20 min and imaged live by confocal microscopy before and after treatment of the same cell at the same focal plane. Repeated independently two times with reproducible results. Scale bars: 10 μm.


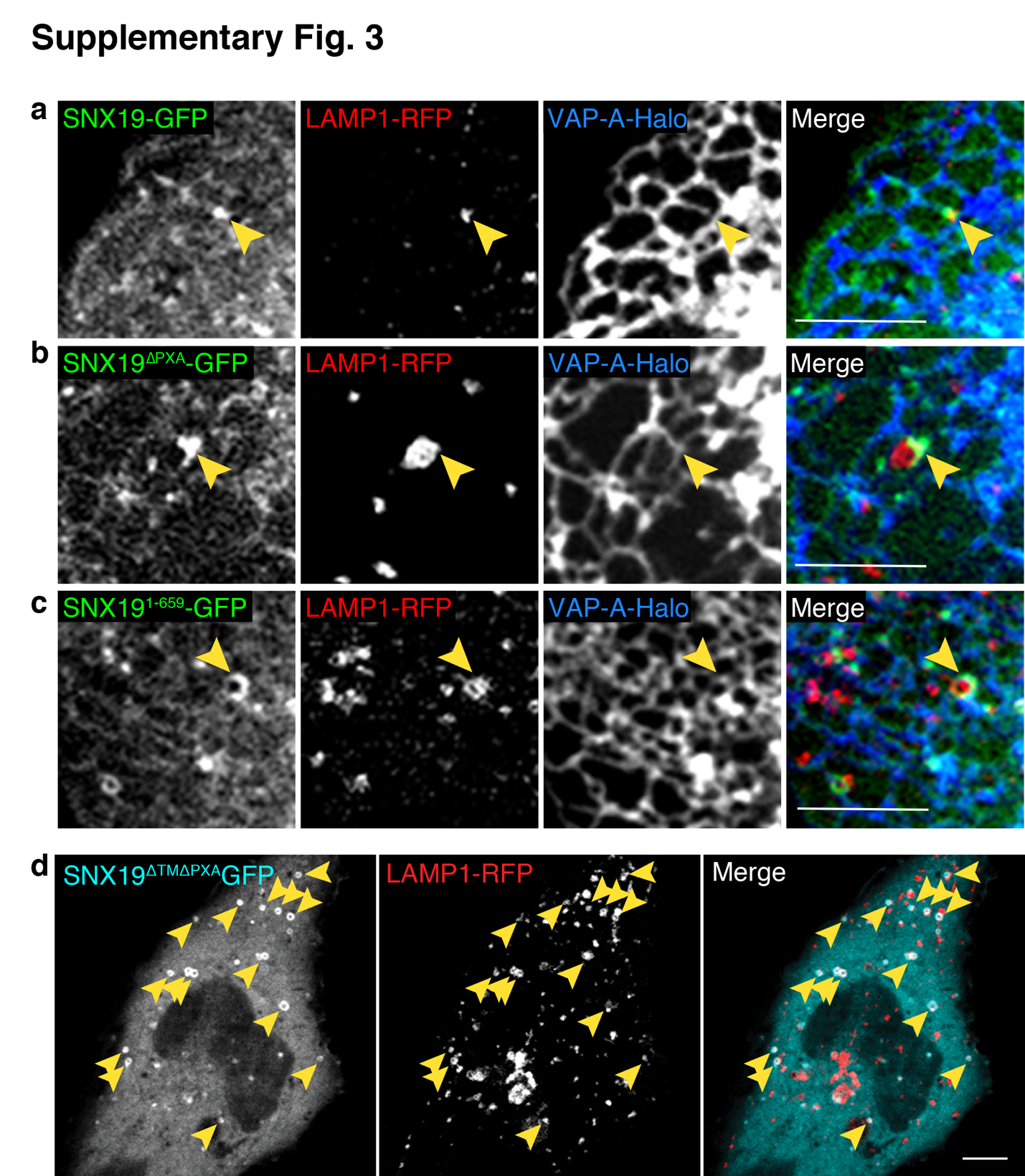


**Supplementary Fig. 3: VAP-A is not concentrated at SNX19-positive ER-EL contacts. a-c,** U-2 OS cells were co-transfected with plasmids encoding LAMP1-RFP, VAP-A-Halo (labeled with Janelia Fluor 646) and SNX19-GFP **(a)**, SNX19^∆PXA^-GFP **(b)** or SNX19^1-659^-GFP **(c)**, and imaged live by confocal microscopy. Arrowheads point to contacts between SNX19 (WT or hypertethers) and ELs. Experiments were independently conducted 3 times. Contact in **b** is also shown in Supplementary Movie 2. **d,** U-2 OS cells were co-transfected with plasmids encoding SNX19^∆TM∆PXA^-GFP and LAMP1-RFP and imaged live by confocal microscopy. Experiment was independently conducted two times and was reproducible. Arrowheads point to SNX19^∆TM∆PXA^-GFP-positive ELs. Scale bars: 10 μm.


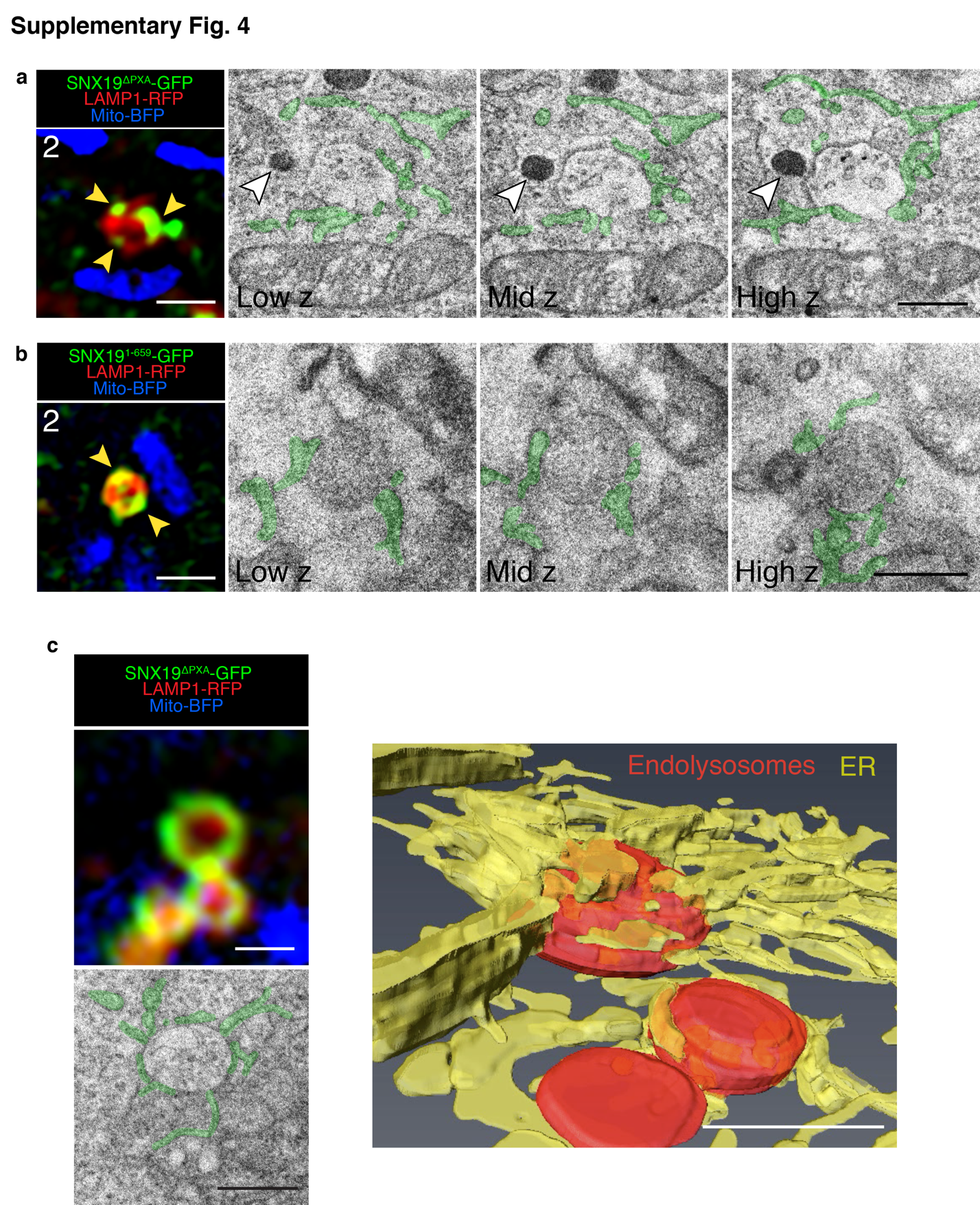


**Supplementary Fig. 4: CLEM imaging of U-2 OS cells expressing SNX19 hypertethers. a** and **b,** Fluorescence micrographs show SIM images (left) and EM images (right) of contacts in box 2 of Fig. 4a **(a)** and box 2 of Fig. 4c **(b)**. Yellow arrowheads point to SNX19^ΔPXA^-GFP **(a)** or SNX19^1-659^-GFP **(b)** enrichment on ELs containing LAMP1-RFP. The green shading in EM images (right) highlights the ER. White arrowheads point to a LD **(a)**. **c,** Three-D reconstruction (right) of SNX19^ΔPXA^-GFP contact with ELs containing LAMP1-RFP (left). The reconstruction was generated from serial EM sections (one mid-Z section shown on the left, bottom, with ER shaded in green) corresponding to the contacts captured in the SIM micrograph (left, top). The CLEM experiment was conducted two times with multiple cells and multiple tethering events captured per condition. Scale bars: 500 nm.

**
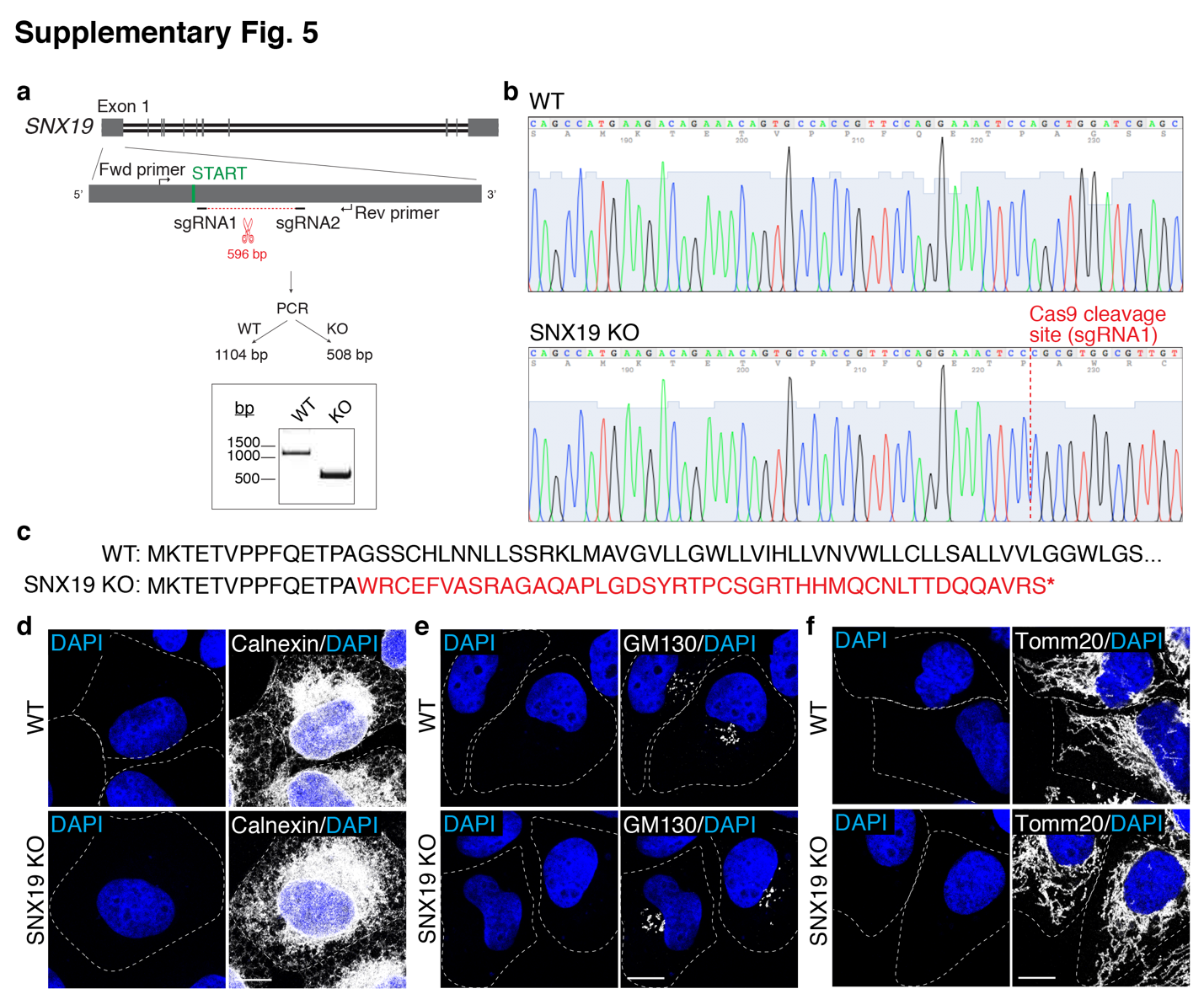
**

**Supplementary Fig. 5: SNX19-KO U-2 OS cells have normal ER, Golgi and mitochondria. a,** SNX19 KO strategy in U-2 OS cells. Bands show PCR products produced with the indicated forward and reverse primers in WT and SNX19-KO cells. PCR screening of this cell line was done once and the bands sequenced. **b,** Sequencing data of WT and SNX19-KO cells of CRISPR-Cas9-edited region, conducted by excising bands from gel in **a**, purifying DNA and using forward primer from **a**. **c**, Expected SNX19 translation in SNX19-KO cells based on sequencing data in **b** (asterisk indicates last residue). **d-f,** WT and SNX19-KO U-2 OS cells were fixed and immunostained for the ER marker calnexin **(d)**, Golgi marker GM130 **(e)** and mitochondrial marker Tomm20 **(f)**. Cell outlines are shown with dashed lines. Experiments independently conducted two times and were reproducible. Scale bars: 10 μm.

**
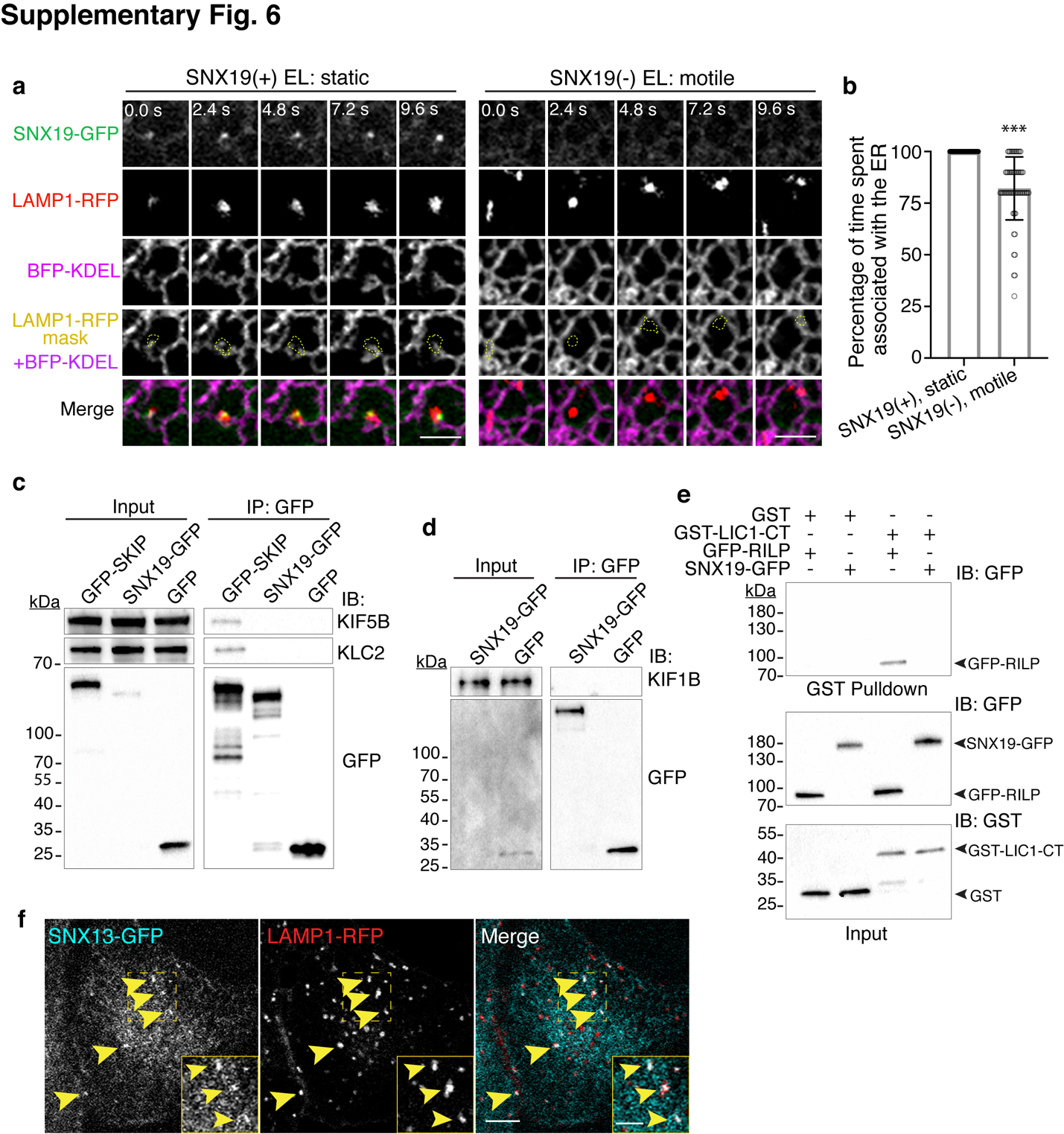
Supplementary Fig. 6: Association of SNX19-positive ELs with the ER and lack of interaction of SNX19 with endolysosomal motor proteins. a,** U-2 OS cells were co-transfected with plasmids encoding SNX19-GFP, LAMP1-RFP and BFP-KDEL and imaged live by confocal fluorescence microscopy. Images are representative close-ups of ELs that either had SNX19-GFP accumulated on them and were static (left) or did not have SNX19-GFP accumulation and were motile (right), over 5 consecutive frames. Yellow dashed lines are tracings of the EL (*i.e.,* LAMP1-RFP mask) which were overlaid on the ER signal (BFP-KDEL). **b,** Quantification of EL-ER association from experiments such as that depicted in **a**. A LAMP1-RFP mask was generated for ELs with or without SNX19 accumulation and the presence of BFP-KDEL signal within the mask was scored over time. The number of frames with BFP-KDEL signal within the mask were divided by the total frames (10 for each EL), multiplied by 100 and represented as a percentage of time that the EL is associated with the ER. This analysis was performed on n=53 and n=64 ELs per condition across 2 independent experiments. Data are represented as mean values +/- SD and significance was determined with a one sample t-test of the SNX19(-), motile condition; *** two-tailed p value<0.001. Source data are provided as a Source Data file. **c,** Co-immunoprecipitation of SNX19-GFP with kinesin-1. HEK293T cells were transfected with plasmids encoding SNX19-GFP, the positive control GFP-SKIP or the negative control GFP; immunoprecipitation (IP) was conducted using GFP-Trap magnetic beads and SDS-PAGE and immunoblotting (IB) were performed to detect the endogenous KIF5B and KLC2 chains of kinesin-1 as well as the GFP tag. The positions of molecular mass markers are indicated on the left. Experiment was independently conducted three times and was reproducible. **d,** Co-immunoprecipitation of SNX19-GFP with kinesin-3 (KIF1B) was performed as in **c** using only plasmids expressing SNX19-GFP or the negative control GFP and immunoblotting to detect endogenous KIF1B and the GFP tag. No positive control was available, as KIF1B is not known to require adaptors for association with organelles. The positions of molecular mass markers are indicated on the left. Experiment was independently conducted two times and was reproducible. **e,** GST pulldown assay of dynein and SNX19-GFP. GST-tagged dynein light intermediate chain 1 C-terminal domain (GST-LIC1-CT) or the negative control GST were immobilized on a glutathione resin and incubated with lysates of HEK293T cells transfected with plasmids expressing SNX19-GFP or the positive control GFP-RILP. SDS-PAGE and immunoblotting (IB) were conducted to detect the GFP- and GST-tagged proteins. The positions of molecular mass markers are indicated on the left. Experiment was independently conducted two times and was reproducible. **f,** U-2 OS cells were co-transfected with plasmids encoding SNX13-GFP plus LAMP1-RFP and imaged live by confocal fluorescence microscopy. Arrowheads point to SNX13-GFP–­LAMP1-RFP double-positive structures. Experiment was independently conducted at least two times and was reproducible. Scale bars: 5 μm in **a** and insets, 10 μm in **f.**

**
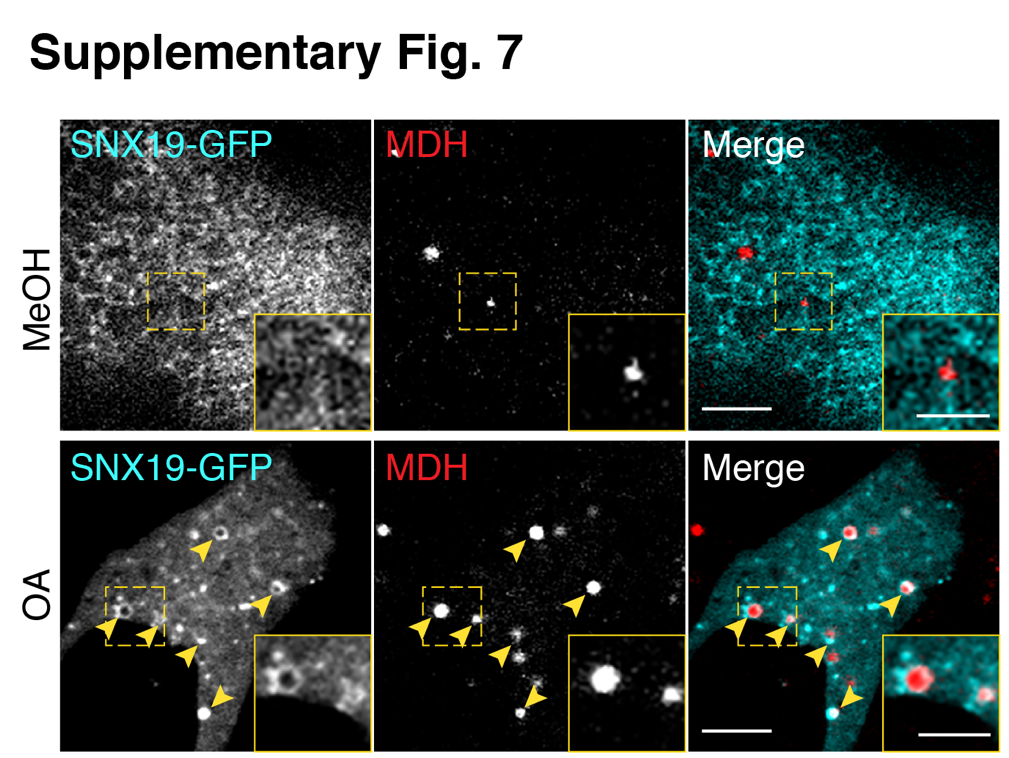
**

**Supplementary Fig. 7: Oleic acid treatment redistributes SNX19 to LDs.** U-2 OS cells transfected with a plasmid encoding SNX19-GFP were treated with either methanol (MeOH) (vehicle control) (top panel) or 200 μM oleic acid (OA) (bottom panel) for 2 h, incubated with monodansylpentane (MDH) for 15 min to label LDs, and imaged live by confocal fluorescence microscopy. Arrowheads point to contacts of SNX19-GFP with LDs. Insets are magnified views of the boxed areas. Experiment was independently conducted three times and was reproducible. Scale bars: 10 μm, 5 μm insets.

**Supplementary Table 1**

| **Primer Name** | **Sequence (5’-3’)** | **Purpose** |
| --- | --- | --- |
| AS191 | Fwd: GATCTCGAGCTCAAGCTTCGATGAAGACAGAAACAGTGC | For cloning SNX19-GFP |
| AS318 | Rev: GTACCGTCGACTGCAGAATTGGAGAGGAGACACCCATCCTC |  |
| AS317 | Fwd: GATCTCGAGCTCAAGCTTCGATGCCATGCCCTGAGGCAGAAAG | For cloning SNX19^∆TM^-GFP |
|  | Rev: AS318 |  |
| AS306 | Fwd: GTACCGCGGGCCCGGGATCCACCGGTCGCCACCATGGCAGAAATCGGTACTG | For cloning SNX19-Halo |
| AS307 | Rev: GATTATGATCTAGAGTCGCGGCCGCTTTAGCCGGAAATCTCGAGCGTC |  |
| AS187 | Fwd: GATCTCGAGCTCAAGCTTCGATGTTAACTGAGGCCAGTC | For cloning SNX13-GFP |
| AS188 | Rev: GTACCGTCGACTGCAGAATTCCTTTTCTGCAAAGAAGG |  |
| AS319 | Fwd: GATCTCGAGCTCAAGCTTCGATGAGAGATCCAGCACCCTGCC | For cloning SNX19 ^∆TM∆PXA^-GFP |
|  | Rev: AS318 |  |
| AS293 | Fwd: CTGTTCCAGGGGCCCCTGGGATCCATGAACCTTCGTATCACTGGCACC | For cloning GST-PX |
| AS294 | Rev: CACGATGCGGCCGCTCGAGCTATTTTTCGAACTGCGGGTGGCTCCAGCCACCGTTCAGAGCAAGGAACTCCTG |  |
|  | Fwd: AS191 | For cloning SNX19^1-659^-GFP |
| AS282 | Rev: GTACCGTCGACTGCAGAATTGGGTTCAGAGCAAGGAACTCC |  |
| AS12 | Fwd: CGGGGATCCGCAGCTGGAAGGCCTGTGGATGCCTCA | For cloning GST-LIC1-CT |
| AS10 | Rev: GCGGCTCGAGTCAAGAAGCTTCTCCTTCCGTAGGAG |  |
| AS254 | Fwd: CCGACCTCTCTCCCCAGGGGATCCGCGGCCGCCACCATGGTGCCCTGGGTGCGG | For cloning SNX14-mNeonGreen |
| AS255 | Rev: CGCCCTTGCTCACGCTAGCCGGGCCCGCGGTACCCATCCAAGATGTCACAGAGG |  |
| AS380 | Fwd: GGACTCAGATCTCGAGCTCAAGCTTCGATGGCGAAACACGAGCAAATC | For cloning VAP-A-Halo |
| AS381 | Rev: TCTGCCATGGTGGCGACCGGCGAGATGAATTTCCCTAGAAAGAATC |  |
| AS298 | Fwd: CTGTGAATCGTCAGTATCGGGAGTTC | For mutagenesis of SNX19-GFP to generate SNX19^R582Q^-GFP |
| AS299 | Rev: CTGTGAATCGTCAGTATCGGGAGTTC |  |
| AS302 | Fwd: CACCTGTCCTCCATGCCCTGAGGCAGATCCAGCACCCTGCCCAGCCAGTG | For mutagenesis of SNX19-GFP to generate SNX19^∆PXA^-GFP |
| AS303 | Rev: CACTGGCTGGGCAGGGTGCTGGATCTGCCTCAGGGCATGGAGGACAGGTG |  |
| Rab5 Halo KI F | Fwd: GAAGGTCTAGTAGAGTTTAAG | For screening Rab5-Halo KI CRISPR-edited cells |
| Rab5 Halo KI R | Rev: GCTGAACAGCTCTTTTCTTC |  |
| AS407 | Fwd: TTGGAGGGCTCTGTTAGTGG | For screening SNX19 KO CRISPR-edited cells |
| AS408 | Rev: TACCTCAGCAATCAGTGGCA |  |

**Supplementary Table 1: List of primers used in this study and their purposes.**
