# Supplementary material for: SNX19 restricts endolysosome motility through contacts with the endoplasmic reticulum
Source: Nat Commun. 2021 Jul 27;12:4552. doi: 10.1038/s41467-021-24709-1 (PMC8316374; doi:10.1038/s41467-021-24709-1)
Supplement: Supplementary file 3 — Description of Additional Supplementary Files [file 41467_2021_24709_MOESM3_ESM.pdf]

Description of Additional Supplementary Files

File Name: Supplementary Movie 1
Description: **SNX19-GFP makes contacts with LAMP1-RFP-positive organelles.** U-2 OS cell from Fig. 1e was co-transfected with plasmids encoding SNX19-GFP and LAMP1-RFP and recorded live. Images were acquired every 0.75 seconds for 58 seconds. Playback is at 30 frames per second. Most SNX19-GFP–LAMP1-RFP puncta are stable and relatively static in this time frame. This video illustrates a rarer instance in which a SNX19-GFP–LAMP1-RFP punctum moves, as another way of demonstrating the association of these markers over time. Scale bar: 10 µm.

File Name: Supplementary Movie 2
Description: **VAP-A is not recruited to ER-EL contacts containing the SNX19$^{\Delta PXA}$ hypertether.** U-2 OS cell from Supplementary Fig. 3b was co-transfected with plasmids encoding SNX19$^{\Delta PXA}$-GFP, LAMP1-RFP and VAP-A-Halo (labeled with Janelia Fluor 646 dye) and recorded live. Yellow arrow points to SNX19$^{\Delta PXA}$-GFP–LAMP1-RFP contact. Images were acquired every 2.6 seconds for 44 seconds. Playback is at 10 frames per second. Scale bar: 10 µm.
