# Supplementary material for: SNX19 restricts endolysosome motility through contacts with the endoplasmic reticulum
Source: Nat Commun. 2021 Jul 27;12:4552. doi: 10.1038/s41467-021-24709-1 (PMC8316374; doi:10.1038/s41467-021-24709-1)
Supplement: Supplementary file 6 — Reporting Summary [file 41467_2021_24709_MOESM6_ESM.pdf]

nature research

Corresponding author(s): Juan S. Bonifacino
Last updated by author(s): Apr 27, 2021

Reporting Summary

Nature Research wishes to improve the reproducibility of the work that we publish. This form provides structure for consistency and transparency in reporting. For further information on Nature Research policies, see our Editorial Policies and the Editorial Policy Checklist.

Statistics

For all statistical analyses, confirm that the following items are present in the figure legend, table legend, main text, or Methods section.

n/a	Confirmed	
☐	☒	The exact sample size (n) for each experimental group/condition, given as a discrete number and unit of measurement
☐	☒	A statement on whether measurements were taken from distinct samples or whether the same sample was measured repeatedly
☐	☒	The statistical test(s) used AND whether they are one- or two-sided<br>*Only common tests should be described solely by name; describe more complex techniques in the Methods section.*
☒	☐	A description of all covariates tested
☒	☐	A description of any assumptions or corrections, such as tests of normality and adjustment for multiple comparisons
☐	☒	A full description of the statistical parameters including central tendency (e.g. means) or other basic estimates (e.g. regression coefficient) AND variation (e.g. standard deviation) or associated estimates of uncertainty (e.g. confidence intervals)
☐	☒	For null hypothesis testing, the test statistic (e.g. F, t, r) with confidence intervals, effect sizes, degrees of freedom and P value noted<br>*Give P values as exact values whenever suitable.*
☒	☐	For Bayesian analysis, information on the choice of priors and Markov chain Monte Carlo settings
☒	☐	For hierarchical and complex designs, identification of the appropriate level for tests and full reporting of outcomes
☒	☐	Estimates of effect sizes (e.g. Cohen's d, Pearson's r), indicating how they were calculated

Our web collection on statistics for biologists contains articles on many of the points above.

Software and code

Policy information about availability of computer code

Data collection	For endolysosome motility tracking, u-track software containing the moment scaling spectrum (MSS) Matlab algorithm was used (Jaqaman et al. Nat Methods 2008, PMID: 18641657) and is publicly available at https://www.utsouthwestern.edu/labs/jaqaman/software/.<br><br>The custom Fiji macro for semi-automation of shell analysis used to measure EL positioning has been deposited on Zenodo (DOI: 10.5281/zenodo.4722031).<br><br>Microscopy images were acquired using Zeiss ZEN Black software version 14.0.18.201.
Data analysis	Microscopy images were analyzed by the open-source software Fiji v1.52p (NIH, Bethesda, MD). Statistical tests were done using the commercially available software Prism v8.3.1 (GraphPad Software, San Diego, CA, USA). For CLEM 3D reconstruction, the commercially available software Amira v6.5.0 (Thermo Scientific, Waltham, MA, USA) was used.

For manuscripts utilizing custom algorithms or software that are central to the research but not yet described in published literature, software must be made available to editors and reviewers. We strongly encourage code deposition in a community repository (e.g. GitHub). See the Nature Research guidelines for submitting code & software for further information.

Data

Policy information about availability of data

All manuscripts must include a data availability statement. This statement should provide the following information, where applicable:

- Accession codes, unique identifiers, or web links for publicly available datasets
- A list of figures that have associated raw data
- A description of any restrictions on data availability

Source data for Figures 1F, 1H, 2D, 2G, 3C, 5B, 5D, 5F, 6B, 6D, 6F, 6H, 6J, 7B, 7E, 7G and Supplementary Figures 1D, 6B will be provided with the paper. Any other data supporting the findings of this study will be available from the corresponding author upon reasonable request.

Field-specific reporting

Please select the one below that is the best fit for your research. If you are not sure, read the appropriate sections before making your selection.

☒ Life sciences ☐ Behavioural & social sciences ☐ Ecological, evolutionary & environmental sciences

For a reference copy of the document with all sections, see nature.com/documents/nr-reporting-summary-flat.pdf

Life sciences study design

All studies must disclose on these points even when the disclosure is negative.

Sample size	No sample-size calculations were performed. Sample size was determined by considering sample sizes used in similar experiments previously reported in the literature (ie. Alpy et al. J Cell Sci 2013, PMID: 24105263) and by our technical ability to maximize sample size. This typically resulted in at least 10 cells quantified per condition per experiment and at least n=3 experiments.
Data exclusions	As stated in methods, endolysosome positioning was quantified in those cells that exhibited a relatively round shape and a centered nucleus as elongated narrow cells were not amenable to the quantification method used ("shell" analysis). These criteria were pre-established and the same criteria were applied to all conditions tested.<br>Occasionally, up to 10% of SNX19-GFP-expressing cells had a cytosolic GFP signal, in which case SNX19-endolysosome contacts could not be quantified in those cells. We attributed this to cleavage of the GFP tag and/or plasmid preparation. Fresh plasmid DNA preps of SNX19-GFP often expressed better and did not exhibit this issue.<br>Additionally, any transfected cells that were expressing excessively high levels of a protein that visibly altered cell morphology or exhibited saturated signal intensities, were excluded from analyses.
Replication	Data were reproducible and quantified experiments were conducted independently at least 3 times to confirm reproducibility. An exception is the EM data in Figure 5. This experiment was conducted 2 times and was reproducible. All other data that did not require quantification were reproduced independently at least 2 times as stated in the individual figure legends.
Randomization	Allocation was not random as this did not apply. All cells were imaged and analyzed in the same way.
Blinding	Blinding was not done as cells were imaged and analyzed in the same way.

Reporting for specific materials, systems and methods

We require information from authors about some types of materials, experimental systems and methods used in many studies. Here, indicate whether each material, system or method listed is relevant to your study. If you are not sure if a list item applies to your research, read the appropriate section before selecting a response.

Materials & experimental systems

n/a	Involved in the study	
☐	☒	Antibodies
☐	☒	Eukaryotic cell lines
☒	☐	Palaeontology and archaeology
☒	☐	Animals and other organisms
☒	☐	Human research participants
☒	☐	Clinical data
☒	☐	Dual use research of concern

Methods

n/a	Involved in the study	
☒	☐	ChIP-seq
☒	☐	Flow cytometry
☒	☐	MRI-based neuroimaging

Antibodies

Antibodies used	Primary antibodies used:<br>mouse anti-calnexin used at 1:500 (Cat# MAB3126, Clone C8.B6, Lot 2919754, Millipore Sigma)

mouse anti-LAMP1 used at 1:500 (DSHB Hybridoma Product Clone H4A3, deposited to the DSHB by J.T. August, J.T and J.E.K. Hildreth)
rabbit anti-LAMP1 used at 1:500 (Cat# 9091, Clone D2D11, Lot 5, Cell Signaling Technology)
rabbit anti-Tomm20 used at 1:1,000 (Cat# ab186734, Clone EPR15581-39, Lot GR32327 12-3, Abcam)
mouse anti-GM130 used at 1:500 (Cat# 610823, Clone 35/GM130, BD Biosciences)
mouse anti-EEA1 used at 1:500 (Cat# 610456, Clone 14/EEA1, Lot 6252701, BD Biosciences)
goat anti-GST-HRP antibody used at 1:2,000 (Cat# GERPN1236, Millipore Sigma)
rabbit anti-Rab5 used at 1:500 (Cat# 3547, Clone C8B1, Lot 7, Cell Signaling Technology)
mouse anti-Halo tag used at 1:1,000 (Cat# G9211, Lot 0000341144, Promega).
rabbit anti-KIF5B used at 1:1000 (Cat# 167429, Clone EPR10276(B), Lot GR3192682-3, Abcam)
rabbit anti-KLC2 used at 1:1000 (Cat# 95881, Lot GR250663-4, Abcam)
rabbit anti-KIF1B used at 1:1000 (Cat# A301-055A, Bethyl Laboratories)
mouse anti-GFP-HRP used at 1:5000 (Cat# 130-091-833, Clone GG4-2C2.12.10, Lot 5180427023, Miltenyi Biotec)

Secondary antibodies used for immunofluorescence microscopy were from Thermo Fisher Scientific and used at 1:2,000:
donkey anti-mouse IgG Alexa Fluor 488 (Cat# A21202, Lot 1796361)
donkey anti-rabbit IgG Alexa Fluor 488 (Cat# A21206, Lot 1796375)
donkey anti-mouse IgG Alexa Fluor 555 (Cat# A31570)
donkey anti-rabbit IgG Alexa Fluor 555 (Cat# A31572, Lot 2088692)
donkey anti-mouse IgG Alexa Fluor 647 (Cat# A31571)
donkey anti-rabbit IgG Alexa Fluor 647 (Cat# A31573, Lot 1903516)
The following secondary antibodies, also purchased from Thermo Fisher Scientific, were used for immunoblotting at 1:10,000:
goat anti-rabbit IgG (H+L) HRP-conjugated (Cat# G-21234)
goat anti-mouse IgG (H+L) HRP-conjugated (Cat# 62-6520)

Validation

All antibodies used were chosen based on suppliers' recommendation for the particular application.
The rabbit anti-LAMP1 additionally was validated by the supplier (Cell Signaling Technology) to work for immunostaining in HeLa cells (same species as our U-2 OS cells).
The anti-Calnexin antibody is currently discontinued. The only species and application validation of this antibody was that we observed the expected structure of the ER in the human cell line U-2 OS by immunofluorescence.
The anti-Tomm20 antibody was shown by the supplier (Abcam) and other publication (DOI: 10.7554/eLife.27860) to co-localize with other known mitochondrial markers in human immunostained cells.
The anti-GM130 antibody was shown by the supplier (BD Biosciences) to immunostain an organelle with the distinct appearance of the Golgi apparatus, in the human cell line WI-38.
In our experiments, immunostaining using the above antibodies showed the expected structures for lysosomes (anti-Lamp1), ER (anti-Calnexin), mitochondria (anti-Tomm20) and Golgi (anti-GM130).

Antibodies used in western blots (Anti- Rab5, Halo-Tag, KIF5B, KLC2, KIF1B, GST and GFP) all showed bands of expected molecular weight.
For species validation, anti-Rab5 was shown by the supplier (Cell Signaling Technology) to produce a band at the expected molecular weight with lysates from the human cell line MCF-7. In addition, our Rab5-Halo knock-in HeLa cells showed an expected higher molecular weight band in the edited cells, corresponding to Halo-tagged endogenous Rab5 protein.
The Halo-Tag antibody was shown by the supplier (Promega) to recognize Halo-Tag fusion proteins based on their correct molecular weight. Like the Rab5 antibody, the Halo-Tag antibody showed an expected higher molecular weight band in our Rab5-Halo knock-in Hela cells, corresponding to Halo-tagged endogenous Rab5 protein. This antibody showed no band in WT unedited HeLa cells.
The KIF5B antibody was species-validated by the supplier (Abcam) using KIF5B KO human HAP1 cells that specifically showed loss of a 110 kDa protein compared to WT HAP1 cells.
For KLC2 and KIF1B antibodies, the suppliers (Abcam and Bethyl Laboratories, respectively) showed by WB and IP that a band at the expected molecular weight was detected with each of these antibodies. IPs with control IgGs did not show any bands with these antibodies.
For the GST and GFP antibodies, the only validation was that our lysates expressing GST or GFP fusion proteins showed bands at expected molecular weights.
The EEA1 antibody was previously shown to partially co-localize with the early/sorting organelle marker transferrin (DOI: 10.1091/mbc.01-07-0380) in tTA-HeLa cells by immunofluorescence.

Eukaryotic cell lines

Policy information about cell lines

Cell line source(s)	The following cell lines were purchased from ATCC: U-2 OS (ATCC HTB-96), HeLa (ATCC CCL-2)<br>The following cell line was purchased from Takara Bio. Inc.: HEK293T (Cat# 632180)
Authentication	Cells authenticated by manufacturer and morphology was assessed by microscopy. Cells were kept frozen at low passage number and thawed fresh after no more than 20 passages.
Mycoplasma contamination	Cells were not directly tested for mycoplasma. Routine staining with DAPI however was conducted to ensure no bacterial or mycoplasma contamination was present.
Commonly misidentified lines<br>(See ICLAC register)	No commonly misidentified lines were used.
